# Delayed Blunt Traumatic Carotid Artery Dissection After a Scooter Accident: A Case Report

**DOI:** 10.5811/cpcem.2022.1.55058

**Published:** 2022-04-25

**Authors:** Robert Rigby, Suneil Agrawal

**Affiliations:** Desert Regional Medical Center, Department of Emergency Medicine, Palm Springs, California

**Keywords:** blunt traumatic injury, carotid artery dissection, CT angiogram neck, case report

## Abstract

**Introduction:**

Traumatic carotid artery dissections (CAD) are rare but produce potentially devastating injuries. Most patients develop symptoms within 72 hours of traumatic injury.

**Case Report:**

We report the case of a 33-year-old, previously healthy male who presented to the emergency department for evaluation of transient, right-sided facial droop with visual changes. His symptoms began 12 days after falling off a scooter. Imaging revealed an extracranial internal CAD.

**Conclusion:**

Symptoms of CAD may present weeks after blunt trauma, making clinical diagnosis difficult. Clinicians must have high suspicion for vascular injury and consider neuroimaging in cervical flexion/extension injuries.

## INTRODUCTION

A carotid artery dissection (CAD) results from tearing in the intimal layer of the artery leading to thrombus formation, wall hematoma, and even lumen occlusion. A dissection occurs spontaneously or traumatically and is classified as extracranial or intracranial. Extracranial arteries are more mobile and thus, hypothetically, more prone to injury. A traumatic dissection may occur from penetrating injury or from blunt injury. There are four fundamental mechanisms of blunt carotid artery injury described: direct application of force to the neck (type I); hyperextension and contralateral rotation of the head and neck (type II); intraoral trauma affecting the carotid artery at the angle of the jaw (type III); or lacerations of the carotid artery resulting from a basilar skull fracture (type IV).^1^

The reported annual incidence rate of CAD is 2.6 to 2.9 per 100,000.^2^ Internal CADs (ICAD) sustained from blunt traumatic injuries are the most common subset of cerebrovascular injuries identified.^2^ There is an increase in cervical artery dissection diagnosis, likely secondary to increased use of computed tomography angiography (CTA) performed in the screening of trauma patients. Recent publications indicate a blunt cerebrovascular injury (BCVI) incidence between 1–2% of in-hospital trauma population and up to 9% of patients with severe head injury.^3^ Vertebral and carotid artery dissections are the most frequent cause of cerebrovascular accidents in the young, with over half presenting with stroke or transient ischemic attacks (TIA).^4^ Typical symptoms of cervical artery dissections include neurologic symptoms involving the anterior or posterior circulation, Horner’s syndrome, cranial or cervical neuropathies, or pulsatile tinnitus. Patients classically have a headache or neck pain at or prior to neurological symptom onset, although typical symptoms may be absent in older patients.^5^ Local or neurological symptom onset is typically within 72 hours of injury, although there are reports of delayed symptoms up to six months.^6^

The incidence of electric scooter injuries has nearly doubled in the United States between 2018 and 2019, and the head is the most common site of injury in scooter-injury patients presenting to the emergency department (ED).^7^ There is one case report of a seven-year-old male who sustained a traumatic vertebral artery dissection and basilar artery occlusion/stroke four days after falling off a scooter.^8^ To our knowledge, there are no case reports of delayed ICAD from a scooter accident.

## CASE REPORT

A 33-year-old Black male with no significant past medical history presented to the ED for evaluation of intermittent, right-sided facial droop and blurred vision. Symptoms were intermittent, lasting approximately 15 minutes at a time, and onset one hour prior to arrival while sitting in a chair. He had a total of three episodes prior to coming into the ED. He had a mild left frontal headache described as dull and non-radiating, with associated lightheadedness, confusion, and palpitations. There were no modifying or alleviating factors. The headache occurred shortly after he noticed a right-sided facial droop in a mirror. His symptoms had completely resolved at the time of initial evaluation.

His right upper extremity was in a splint because of an injury to his wrist sustained 12 days prior after falling off an electric scooter. He denied current arm pain. He denied hitting his head or loss of consciousness during the scooter accident. He denied neck or back pain but stated he may have “whipped” his neck back and forth during the fall. He was not on blood thinners. He denied weakness or numbness/tingling in his arms or legs. Vitals signs were stable with a temperature of 37° Celsius, blood pressure of 128/67 millimeters of mercury, heart rate of 58 beats per minute, respiratory rate of 20 breaths per minute, and an oxygen saturation of 97% on room air. Initial physical examination demonstrated no neurological deficits.

There was no Marfanoid habitus, or evidence of Horner’s syndrome or cranial nerve palsies. He had no dysmetria, and he ambulated normally without ataxia. The motor strength of his right upper extremity was difficult to fully assess as he had a splint in place, but he had 5/5 motor strength in his proximal and distal upper and lower extremities bilaterally. His sensation to light touch appeared intact in all extremities. Other than a splint to his right upper extremity, there was no evidence of trauma on examination.

**Image 1:**
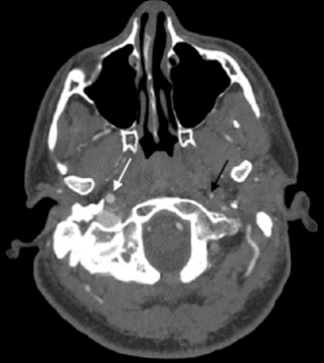
Axial computed tomography demonstrating significant stenosis of the left internal carotid artery (black arrow) vs the patent right internal carotid artery (white arrow).

**Image 2:**
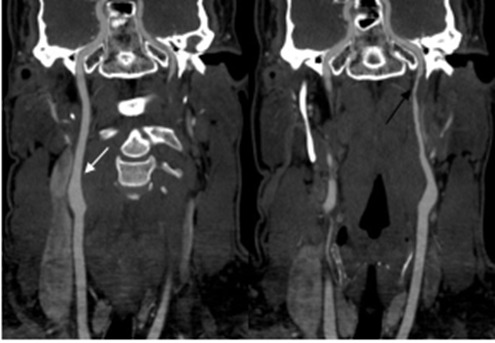
Coronal computed tomography demonstrating patent right internal carotid artery (white arrow) and the stenosis of the left internal carotid artery depicting the “carotid string sign” (black arrow).

An electrocardiogram revealed normal sinus rhythm without abnormalities. Laboratory work including a complete blood count, basic metabolic panel, and troponin were all within normal limits. Neuroimaging was performed and he had a normal computed tomography (CT) of his head without contrast. The CT angiogram (CTA) of the neck demonstrated significant stenosis extending distal to the left carotid bulb to near the skull base with a carotid string sign (Image 1 and 2), consistent with ICAD. A carotid string sign is produced by a stenotic, diminished, weak antegrade flow of contrast material in the internal carotid artery. 16

**Image 3:**
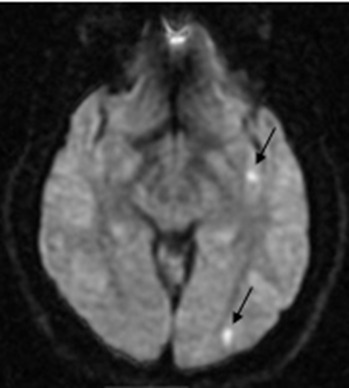
Diffusion-weighted magnetic resonance imaging demonstrating multiple small acute infarcts in the temporal and occipital lobes of the left hemisphere (black arrows). Acute infarcts located in the frontal and parietal region of the left hemisphere are not shown.

He developed very subtle right-sided facial droop, lightheadedness, and a left frontal headache after imaging was performed. Magnetic resonance imaging (MRI) of the brain demonstrated multiple foci of acute infarct involving the left hemisphere (Image 3).

Vascular surgery and neurology were consulted. He was admitted to the telemetry unit of the hospital. Intravenous heparin and oral rosuvastatin were started inpatient. His symptoms resolved completely, and he had an unremarkable hospital stay. He had a normal transthoracic echocardiogram with bubble study performed inpatient. He was discharged two days later on rosuvastatin and apixaban. He was asymptomatic at a six-month phone call follow-up and had normal appearance of the left internal carotid artery on imaging one year later.

**CPC-EM Capsule**

What do we already know about this clinical entity?

*Carotid artery dissection (CAD) can be caused by blunt trauma and is the most frequent cause of cerebrovascular accident in the young*.

What makes this presentation of disease reportable?

*This case highlights a man with a CAD resulting in a transient ischemic attack weeks after a minor mechanism scooter accident*.

What is the major learning point?

*Carotid artery dissection can occur after a minor whiplash mechanism and can present with delayed neurologic deficits*.

How might this improve emergency medicine practice?

*Patients presenting to the emergency department with flexion/extension injury should be considered for computed tomography angiogram neck to evaluate for CAD, although guidelines make no absolute recommendations*.

## DISCUSSION

Carotid artery dissections sustained from blunt traumatic injuries may have a delayed diagnosis given vast symptoms, and some may only become symptomatic and diagnosed once neurological symptoms ensue, as in this case. This patient likely sustained a type II CAD secondary to a whiplash injury resulting in multiple TIAs. The patient was examined by an unknown medical professional the day of his scooter accident and was diagnosed with a right wrist injury. He was placed in a splint; however, he had no neurological or physical complaints until his visit to the ED 12 days later. He specifically denied neck or back pain following the event. It is possible he experienced subtle neck discomfort at the time of injury with a distracting right upper extremity injury, but this was never reported. Given the recent trauma with an adequate mechanism to explain his arterial injury, his dissection was likely traumatic rather than spontaneous, although categorization may be difficult to determine, especially when the initial symptom onset is delayed from the time of arterial injury. Nonetheless, many case reports in the literature demonstrating a delayed presentation of CAD describe a delayed diagnosis, rather than delayed symptom onset.^9^,^10^

A delay in diagnosis before development of neurological symptoms ensue could be prevented with universal screening for BCVI with CTA of the neck. Recent literature supports the application of expanded Denver criteria and supports the liberal use of CTA in trauma patients, as up to 30% of BCVI do not meet screening criteria.^11^,^12^ Expanded Denver criteria recommends CTA if there are signs/symptoms or risk factors for BVCI, which include one or more of the following: arterial hemorrhage from the neck/nose/mouth; cervical bruit in patients less than 50 years old; expanding cervical hematoma; a focal neurological deficit; a neurological exam incongruous with head CT findings; or stroke on secondary CT.

Risk factors for BVCI include a high-energy transfer mechanism with any of the following: Le Fort II or III injury; mandible fracture; complex skull, basilar skull, or occipital skull fracture; severe traumatic brain injury (TBI) with Glasgow Coma Scale <6; cervical spine fracture; subluxation or ligamentous injury at any level; near hanging with anoxic brain injury; seat belt abrasion with significant swelling; pain or altered mental status; TBI with thoracic injury; scalp degloving; thoracic vascular injury; blunt cardiac rupture; or upper rib fracture.^3^

A 2020 study by Leichtle et al showed that adopting universal screening for BCVI with CTA of the neck yielded a diagnosis of BCVI in nearly 20% of patients who would have gone undiagnosed using the most sensitive and extensive screening criteria.^13^ Recent practice management guidelines from the Eastern Association for the Surgery of Trauma (EAST) recommend screening CTA of the neck in high-risk cervical spine injuries and conditionally recommend screening CTA of the neck in low-risk cervical spine injuries.^12^ A high-risk cervical spine injury includes upper cervical spine (C1-C3) fractures, subluxation, and cervical spine fractures that extend into the transverse foramen. Low-risk cervical spine fracture patterns or injuries include any cervical spine injury. Given the incidence of BCVI in low-risk cervical spine injuries ranging from 2–9%, EAST guidelines recommend performing CTA on a case-by-case basis.^12^ Given the mechanism of injury in the patient presented here, there was justification for performing a CTA of the neck during his initial medical visit, although none of the guidelines absolutely required it. If the imaging had been performed, an early diagnosis of CAD may have been made, although his outcome may not have changed.

The patient presented here had an MRI demonstrating multiple tiny acute infarcts to all lobes of the left hemisphere of the brain. It is more common for an ICAD to produce an arterial occlusion by an embolization of a local thrombus rather than the stenosis produced by the dissection. Carotid arteries supply the anterior circulation of the brain, and vertebral arteries predominately supply the posterior circulation of the brain. A fetal posterior cerebral artery (PCA) is present in approximately 20–30% of people and occurs when the PCA partially or fully originates from the internal carotid artery.^14^ This patient had a partial fetal PCA responsible for the infarct to the left occipital lobe.

Specific treatment guidelines of BCVI continue to be controversial, but it is recommended to initiate antithrombotic therapy, either an anticoagulant or antiplatelet, as soon as considered safe, even in the setting of severe head injury or other solid organ injury.^12^ There is a paucity of high-quality evidence guiding specific choice of medical treatment. Some authors recommend low-molecular weight heparin at an antithrombotic dose initially, transitioning to aspirin when feasible.^3^ Routine stenting as treatment is rarely necessary as the potential harms often outweigh the potential benefits.^12^

## CONCLUSION

Traumatic blunt cerebrovascular injuries are a potentially devastating but rare injury. Strokes after a carotid dissection are associated with a mortality rate of 25% and morbidity of 38%.^15^ The integration of CTA head and neck into polytrauma protocols is becoming more common, but there must still be a high clinical suspicion for BCVI in what appear to be minor whiplash injuries. This case supports the conditional recommendation of the recent EAST guidelines and the recent 2020 study by Leichtle et al regarding universal screening for BCVI with CTA neck in all trauma patients. Patients presenting to the ED for evaluation of a flexion/extension cervical injury should be informed of potentially life-threatening and neurological sequelae and provided strict precautions to return to the ED if initial vascular imaging is not obtained.

## References

[1] Crissey MM, Bernstein EF (1974). Delayed presentation of carotid intimal tear following blunt craniocervical trauma. Surgery.

[2] Blum CA, Yaghi S (2015). Cervical artery dissection: a review of the epidemiology, pathophysiology, treatment, and outcome. Arch Neurosci.

[3] Brommeland T, Helseth E, Aarhus M (2018). Best practice guidelines for blunt cerebrovascular injury (BCVI). Scand J Trauma Resusc Emerg Med.

[4] Morris NA, Merkler AE, Gialdini G (2017). Timing of incident stroke risk after cervical artery dissection presenting without ischemia. Stroke.

[5] Traenka C, Dougoud D, Simonetti BG (2017). Cervical artery dissection in patients ≥60 years often painless, few mechanical triggers. Neurology.

[6] Pozzati E, Giuliani G, Poppi M (1989). Blunt traumatic carotid dissection with delayed symptoms. Stroke.

[7] Farley KX, Aizpuru M, Wilson JM (2020). Estimated incidence of electric scooter injuries in the US from 2014 to 2019. JAMA Netw Open.

[8] Phung B, Shah T (2019). Traumatic vertebral artery dissection and basilar artery occlusion/stroke in a 7-year-old child: a case report. J Pediatr Intensive Care.

[9] Edmundson SP, Hirpara KM, Ryan RS (2009). Delayed presentation of carotid artery dissection following major orthopaedic trauma resulting in dense hemiparesis. J Bone Joint Surg.

[10] Pampin JB, Tamayo NM, Fonseca RH (2002). Delayed presentation of carotid dissection, cerebral ischemia, and infarction following blunt trauma: two cases. J Clin Forensic Med.

[11] Bensch FV, Varjonen EA, Pyhältö TT (2019). Augmenting Denver criteria yields increased BCVI detection, with screening showing markedly increased risk for subsequent ischemic stroke. Emerg Radiol.

[12] Kim DY, Biffl W, Bokhari F (2020). Evaluation and management of blunt cerebrovascular injury: a practice management guideline from the Eastern Association for Surgery of Trauma. J Trauma Acute Care Surg.

[13] Leichtle SW, Banerjee D, Schrader R (2020). Blunt cerebrovascular injury: the case for universal screening. J Trauma Acute Care Surg.

[14] Christoph DH, Souza-Lima F, Saporta MA (2009). Isolated posterior cerebral artery infarction caused by carotid artery dissection. Arch Neurol.

[15] Schicho A, Luerken L, Meier R (2018). Incidence of traumatic carotid and vertebral artery dissections: results of cervical vessel computed tomography angiogram as a mandatory scan component in severely injured patients. Ther Clin Risk Manag.

[16] Kashyap V, Clair D (2006). Carotid string sign. J Vasc Surg.

